# Challenges faced by women in the diagnosis and management of polycystic ovary syndrome: a qualitative study

**DOI:** 10.3389/fmed.2026.1758959

**Published:** 2026-06-03

**Authors:** Merve Yilmaz Menek, Merve Sen, Merve Pehlivan

**Affiliations:** 1 Department of Physiotherapy and Rehabilitation, Faculty of Health Sciences, Istanbul Medipol University, Istanbul, Türkiye; 2 Department of Physiotherapy and Rehabilitation, Faculty of Health Sciences, Bursa Uludag University, Bursa, Türkiye; 3 Department of Midwifery, Faculty of Health Sciences, Istanbul Medipol University, Istanbul, Türkiye; 4 Department of Nutrition and Dietetics, Faculty of Health Sciences, Istanbul Medipol University, Istanbul, Türkiye

**Keywords:** challenges, concerns, diagnosis, management, polycystic ovary syndrome, women’s health

## Abstract

**Objective:**

This study aims to examine the challenges experienced by women in the diagnosis and management of PCOS.

**Methods:**

This study is qualitative and phenomenological. The study included 19 women with PCOS aged over 18 years. The data were collected via a semistructured interview guide with open-ended questions. Content analysis of the transcripts was performed with the MaxQDA Analytics Pro 2024 program to analyze the qualitative data.

**Results:**

The participants had an average age of 23.1 years; 42.1% were overweight or obese, with an average BMI of 24.2. Six main themes were identified: PCOS diagnosis experiences, treatment-related changes, management strategies, experienced challenges, symptoms and coping approaches, and uncertainties and concerns. Participants described diagnosis and treatment as emotionally demanding processes shaped by insufficient information, uncertainty, limited multidisciplinary support, appearance-related distress, fertility concerns, and self-directed coping strategies. Many women reported managing PCOS largely through self-directed strategies, such as medication use, dietary changes, physical activity, cosmetic procedures, and informal information seeking, often without structured multidisciplinary support.

**Conclusion:**

Women had difficulty accepting the diagnosis and experienced feelings of disappointment and anxiety. During this period, rejection of treatment, feelings of uncertainty, and a lack of information and support were commonly reported. A multidisciplinary team should be recommended to provide women with comprehensive information, education, and counseling services.

## 1 Introduction

Polycystic ovary syndrome (PCOS) is a lifelong endocrine disorder that affects 10–15% of women (1). and represents the most common endocrine pathology in reproductive-aged females globally (2). Its multifactorial nature and heterogeneous presentation contribute to diagnostic challenges and variability in clinical management (3). PCOS is characterized by menstrual irregularities, hyperandrogenism, metabolic dysfunction, infertility, and significant psychosocial burden, including anxiety, depression, and body image dissatisfaction (4).

Despite updated diagnostic frameworks such as the NIH, Rotterdam, and AE-PCOS criteria, inconsistencies in clinical application continue to result in delayed diagnoses, inadequate information provision, and patient dissatisfaction (5, 6). These challenges are compounded by limited standardized guidance and communication gaps between healthcare providers and patients. Significant dissatisfaction with the diagnostic procedure, information provided, and recommended treatment has also been reported by women with PCOS (7).

Management primarily aims to optimize hormonal, metabolic, and psychological health. Lifestyle modification including individualized nutrition and physical activity is considered first-line therapy and has shown benefits independent of weight loss, particularly in improving insulin sensitivity, ovulatory function, and psychological well-being (8, 9). However, international guidelines highlight barriers to adherence and emphasize the importance of patient-centered, sustainable, and multidisciplinary approaches (10).

Previous research has consistently demonstrated that women with PCOS frequently experience delayed diagnosis, insufficient information, and dissatisfaction with healthcare encounters (7). Qualitative studies have highlighted that these challenges extend beyond clinical symptoms, encompassing feelings of uncertainty, frustration, and lack of support during interactions with healthcare providers (11, 12). In particular, women often report that their concerns are minimized, their symptoms are normalized, and they are expected to self-manage complex lifestyle and treatment decisions with limited professional guidance (11).

Importantly, the experience of PCOS cannot be fully understood solely through its clinical manifestations. Rather, it is embedded within broader sociocultural contexts related to gender roles, body image, fertility expectations, and social norms. Visible symptoms such as hirsutism, acne, and weight gain may lead to body dissatisfaction, stigma, and social withdrawal, while concerns about infertility may generate anxiety and pressure related to future family roles (13, 14). These experiences suggest that PCOS represents not only a medical condition but also a gendered and socially constructed health experience shaped by interpersonal relationships and cultural expectations.

Although a growing body of literature has examined barriers to diagnosis and management, much of the existing research has focused on identifying challenges in a descriptive manner. There remains a need for more interpretive qualitative work that explores how women make sense of these experiences within their sociocultural environments. In particular, limited evidence exists regarding how women in Turkey experience PCOS in relation to family influence, social expectations, stigma, and reproductive concerns, and how these factors shape their engagement with healthcare and self-management strategies.

Therefore, this qualitative study aims to explore how women with PCOS in Turkey interpret and make sense of their diagnosis and management experiences, with a particular focus on the emotional, social, and relational meanings attached to delayed diagnosis, treatment navigation, body-related changes, and fertility-related uncertainty.

## 2 Materials and methods

### 2.1 Study design

This study employed a qualitative descriptive design with a phenomenological orientation to explore women’s experiences of receiving a diagnosis of polycystic ovary syndrome and managing the condition over time. A qualitative descriptive approach was considered appropriate to capture participants’ lived experiences and the meanings they attributed to diagnosis, treatment, and self-management processes without aiming to develop a formal phenomenological essence or theory.

The study focused on identifying recurring patterns of meaning, emotional responses, and perceived challenges embedded in women’s narratives, rather than quantifying prevalence or testing predefined hypotheses.

### 2.2 Setting and participants

The study was conducted between May and September 2024 at Istanbul Medipol University in Istanbul, Turkey. Participants were recruited using purposive sampling to ensure the inclusion of women who were able to provide rich and reflective accounts of living with PCOS. The inclusion criteria were being aged 18 years or older, having received a diagnosis of PCOS from a gynecologist, at least one year having elapsed since the diagnosis, having no comorbid endocrine disease, and being fluent in Turkish. Women who were pregnant, had non-classical congenital adrenal hyperplasia, or declined participation for any reason were excluded. A total of 19 women participated in the study. Data collection continued until data saturation was achieved, defined as the point at which no new meanings or experiential patterns emerged from successive interviews.

The sample size was determined based on principles of qualitative research rather than statistical representativeness. Data collection continued until data saturation was reached, defined as the point at which no new codes, meanings, or experiential patterns emerged. The final sample of 19 participants was therefore considered sufficient to provide rich and in-depth insights into women’s experiences with PCOS.

### 2.3 Data collection

Data were collected through face-to-face semi-structured interviews, supported by a sociodemographic information form. The interview guide was developed based on existing qualitative literature on PCOS and refined through expert feedback from researchers experienced in women’s health and qualitative research. A pilot interview was conducted with three women (not included in the final sample) to ensure clarity and comprehensibility of the questions.

The interview questions addressed:

- Experiences of PCOS diagnosis
- Perceived changes during treatment
- Management strategies
- Experienced challenges
- Symptoms and coping approaches
- Uncertainties and concerns related to PCOS

Although the interview guide included questions addressing specific domains such as diet, physical activity, and emotional concerns, participants were encouraged to elaborate freely and introduce issues they considered personally meaningful. Interviews were conducted in a private setting to ensure confidentiality and lasted approximately 40 min. All interviews were audio-recorded with written and verbal consent and transcribed verbatim.

### 2.4 Data analysis

Qualitative data were analyzed using inductive content analysis, supported by MAXQDA Analytics Pro 2024 software. The analysis followed a constant comparative approach. The coding process was conducted in several stages. First, two researchers independently read all transcripts several times to become familiar with the data. Second, meaningful units related to women’s experiences of diagnosis, treatment, symptoms, coping, and uncertainty were identified. Third, initial codes were generated inductively from participants’ expressions. Similar codes were then compared and clustered into subcodes according to conceptual similarity. In the next stage, related subcodes were grouped into broader categories and higher-order themes. The code–subcode–theme structure was repeatedly reviewed by the research team to ensure that the final thematic framework reflected participants’ narratives rather than only the interview guide.

To enhance coding reliability, two researchers independently coded the transcripts and then compared their coding decisions. Disagreements regarding code and subcode definitions were discussed until consensus was reached. A preliminary codebook including code names, subcode definitions, inclusion criteria, and representative quotations was developed. The final codebook was reviewed by all members of the research team. Intercoder agreement was calculated using the Miles and Huberman formula and was found to be 85.87%, indicating acceptable coding consistency. The study reporting followed the Consolidated Criteria for Reporting Qualitative Research (COREQ) checklist.

### 2.5 Researcher reflexivity

The research team consisted of investigators from women’s health and diseases nursing, physiotherapy, and nutrition disciplines. This multidisciplinary composition informed the interpretation of participants’ accounts related to bodily experiences, lifestyle management, and emotional well-being. To minimize disciplinary bias and researcher dominance, reflexive discussions were held throughout the analysis process. Analytical decisions were made collaboratively, with attention paid to ensuring that themes reflected participants’ voices rather than the structure of the interview guide.

### 2.6 Ethical considerations

Ethical approval was obtained from the Istanbul Medipol University Non-Interventional Ethics Committee (file no: E-10840098-202.3.02-621; decision no: 49; Date: 18 January 2024). Written informed consent was obtained from all participants prior to data collection. Confidentiality and anonymity were ensured by assigning numerical codes to participants and removing identifying information from transcripts.

## 3 Results

The findings of this study are based on in-depth interviews with 19 women diagnosed with polycystic ovary syndrome. Participants described diverse and often complex experiences related to receiving a diagnosis, navigating treatment pathways, and managing the condition over time. The results are presented thematically, reflecting shared patterns of meaning across women’s narratives while preserving individual experiential nuances.

Sociodemographic characteristics of the participants are summarized in Table 1. Briefly, participants ranged in age from 18 to 31 years, with varied educational, employment, and health-related backgrounds, situating their PCOS experiences within different life contexts.

**Table 1:** Sociodemographic characteristics of the participants.

Participant	Age	BMI	Educational level	Employment status	Socio-economic status	Smoking status
P1	22	19.5	High school	Unemployed	Income = expenses	Does not smoke
P2	23	25.9	High school	Unemployed	Income = expenses	Smokes
P3	27	19.1	High school	Unemployed	Income = expenses	Does not smoke
P4	21	33.2	High school	Unemployed	Income>expenses	Does not smoke
P5	20	23.5	University	Employed	Income<expenses	Does not smoke
P6	22	22.6	High school	Unemployed	Income = expenses	Smokes
P7	20	23.8	High school	Unemployed	Income = expenses	Does not smoke
P8	19	24	High school	Unemployed	Income = expenses	Does not smoke
P9	21	21.1	High school	Unemployed	Income = expenses	Does not smoke
P10	20	18.9	University	Employed	Income = expenses	Smokes
P11	21	20.4	High school	Unemployed	Income>expenses	Does not smoke
P12	21	25.2	High school	Unemployed	Income>expenses	Does not smoke
P13	31	19.3	Graduate school	Employed	Income>expenses	Does not smoke
P14	30	19.6	Graduate school	Employed	Income>expenses	Does not smoke
P15	29	32	Graduate school	Employed	Income>expenses	Does not smoke
P16	21	29	High school	Unemployed	Income = expenses	Does not smoke
P17	21	25.3	High school	Unemployed	Income = expenses	Smokes
P18	27	32.7	University	Employed	Income = expenses	Does not smoke
P19	23	26.2	High school	Unemployed	Income = expenses	Does not smoke

### 3.1 Results related to the PCOS diagnosis process

**Figure 1:**
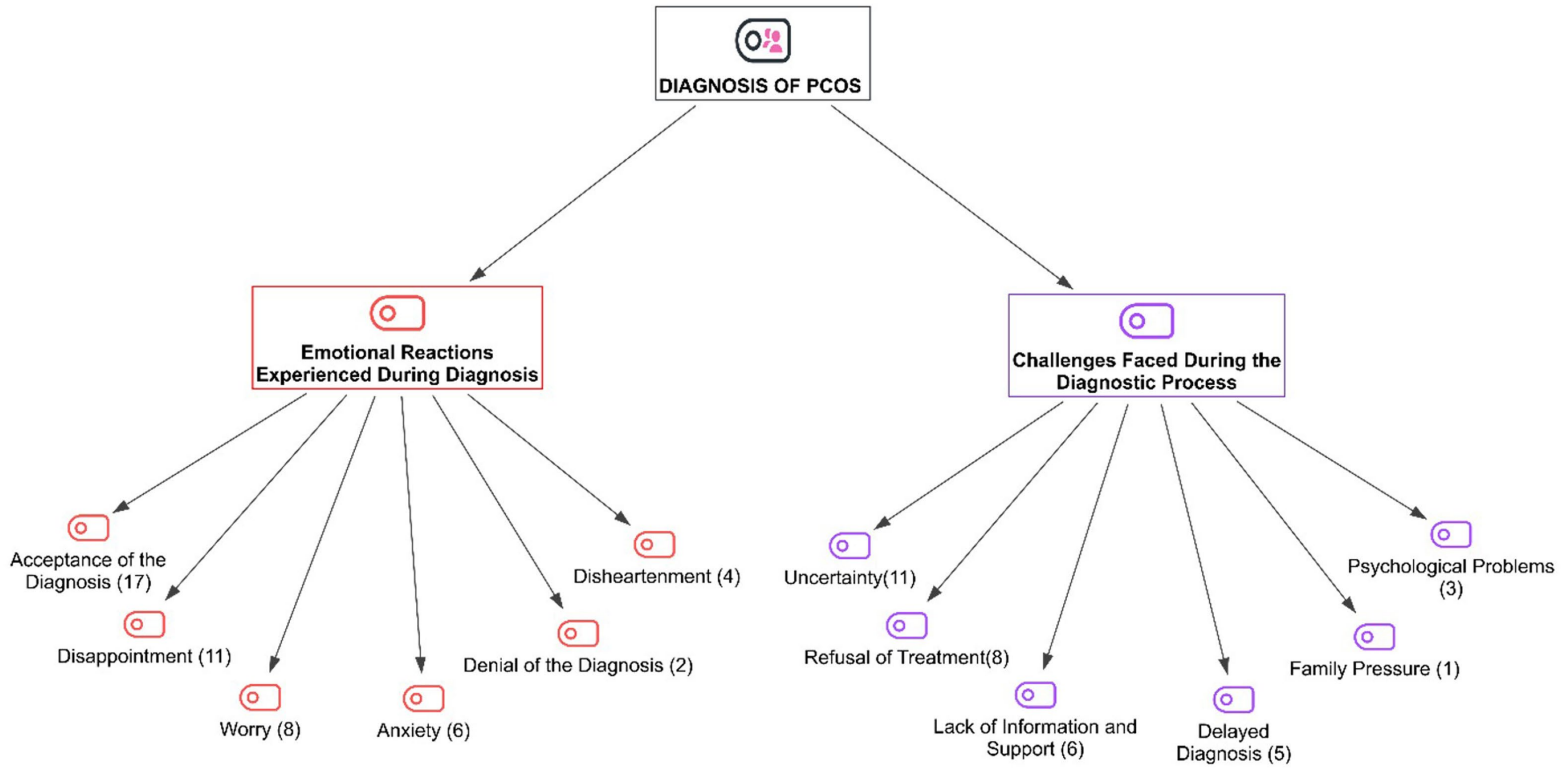
Hierarchical code-subcode model of the PCOS diagnosis process.

Women described the PCOS diagnostic process as a complex and emotionally demanding experience that extended beyond the moment of receiving a medical label. Diagnosis was often framed as a period marked by uncertainty, delayed recognition, and feelings of being insufficiently acknowledged within healthcare encounters. For many participants, the diagnostic phase represented a disruption of bodily certainty and initiated concerns related to health, femininity, and future fertility (Figure 1).

#### 3.1.1 Emotional reactions experienced during the diagnosis process

Participants expressed diverse emotional reactions during the diagnostic process, including acceptance, disappointment, worry, anxiety, denial, and discouragement. Acceptance of the diagnosis was, in some cases, conditional and closely associated with temporary symptom improvement rather than long-term understanding of PCOS as a chronic condition (Figure 1).

*“It started in high school. I used medication for about six months, and when my cycle became regular, I stopped. Now I only go for check-ups occasionally.” (P9).*

Similarly, another participant linked acceptance to symptomatic relief following pharmacological treatment:

*“I went to the doctor because of irregular periods. I started birth control pills, and they helped with my symptoms.” (P11)*

Alongside acceptance, feelings of disappointment and emotional distress frequently emerged when participants perceived that their symptoms were minimized or normalized by healthcare professionals, particularly among younger and unmarried women.

*“Because I’m young and single, especially not sexually active, I wasn’t taken seriously. They kept saying, ‘You’re young, this is normal.’” (P2)*

Such experiences contributed to heightened worry and anxiety, as participants felt uncertain about whether their symptoms were adequately understood or addressed.

*“I started treatment, but the medication caused severe side effects. I couldn’t continue because it made everything worse, especially the bleeding and pain.” (P1)*

For some women, initial emotional responses also included elements of denial or minimization, reflected in discontinuing follow-up or treatment once symptoms temporarily subsided.

*“When my periods improved, I thought maybe it wasn’t that serious, so I stopped the medication.” (P9)*

These accounts suggest that emotional reactions during diagnosis were shaped not only by physical symptoms but also by age- and gender-based expectations within clinical interactions, influencing how seriously women felt their concerns were considered (Figure 1).

#### 3.1.2 Challenges encountered during the diagnosis process

Women reported multiple challenges throughout the diagnostic process, including delayed diagnosis, lack of clear and consistent information, uncertainty regarding disease progression, and limited guidance on treatment options. These challenges often led to confusion and hindered trust in healthcare services.

*“They examined me and said to try medication for three months, then another six months. But nothing changed, and now I don’t even know what my current condition is.” (P4)*

Uncertainty regarding treatment necessity and outcomes frequently resulted in treatment refusal or discontinuation.

*“The medication caused emotional ups and downs, so I decided to stop using it.” (P16)*

In addition to healthcare-related challenges, family influence emerged as a significant contextual factor shaping diagnostic experiences. Some participants described family members discouraging continued treatment, which contributed to early interruption of medical care.

*“The doctor prescribed medication, and I started taking it, but my family didn’t want me to continue, so I stopped after two months.” (P5)*

Overall, the diagnostic phase was characterized by emotional vulnerability, informational gaps, limited support, and emerging feelings of discouragement. These early diagnostic experiences appeared to shape women’s subsequent engagement with treatment, healthcare providers, and long-term PCOS management strategies (Figure 1).

### 3.2 Changes experienced during the treatment process

**Figure 2:**
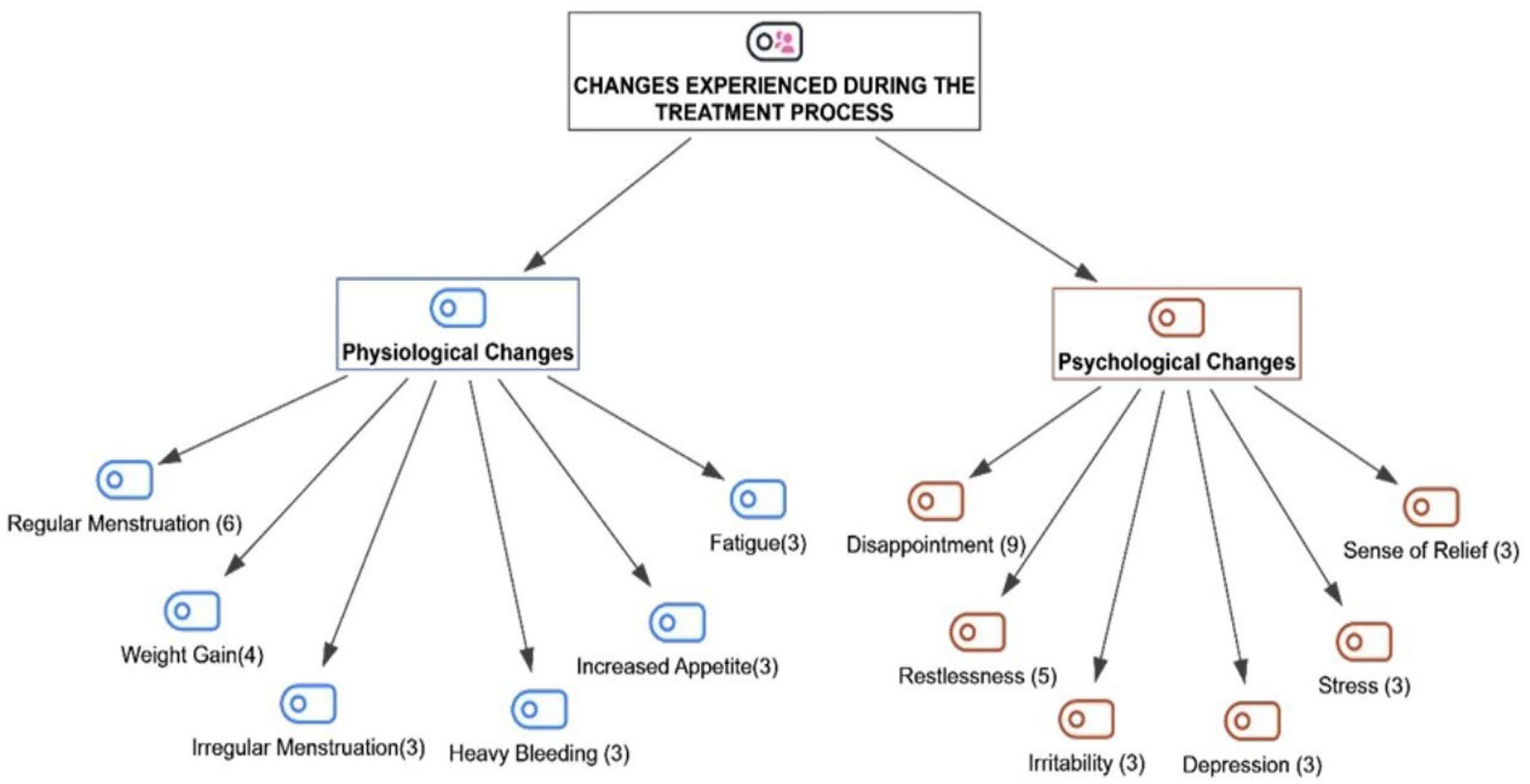
Hierarchical code–subcode model of changes experienced during the treatment process.

Women reported experiencing both physiological and psychological changes during the treatment process, which were often described as fluctuating, unpredictable, and closely intertwined. While some participants reported partial symptom improvement, others emphasized persistent or new challenges that complicated their treatment experiences. These changes frequently shaped women’s motivation, emotional well-being, and perceptions of treatment effectiveness (Figure 2).

#### 3.2.1 Physiological changes experienced during treatment

Participants described a range of physical changes during treatment, most commonly related to menstrual patterns, body weight, bleeding, and fatigue. For some women, treatment initially led to improvements in menstrual regularity, which was perceived as a positive outcome and a sign of treatment effectiveness (Figure 2).

*“During treatment, my menstrual cycle was slightly better, with delays of around four to five days.” (P5)*

Similarly, another participant reported resolution of previously irregular menstruation:

*“My problem with menstrual irregularity was resolved, and it became regular.” (P12)*

However, these improvements were not universal or consistently sustained. Several participants described ongoing irregular menstruation, heavy bleeding, or the emergence of additional physical symptoms during treatment, which contributed to dissatisfaction and uncertainty.

*“During the treatment, I experienced a lot of menstrual irregularity, excessive bleeding, and nausea.” (P1)*

Changes in body weight and appetite also emerged as salient physical experiences. While some participants viewed weight gain as manageable, others found it distressing, particularly when associated with increased appetite or changes in body fat distribution.

*“The medications increased my appetite a lot, especially causing more fat accumulation around my waist.” (P13)*

These physical changes often intensified women’s vigilance toward their bodies and reinforced feelings of being caught in a cyclical process of symptom management.

#### 3.2.2 Psychological changes experienced during treatment

In addition to physical changes, participants described notable psychological and emotional shifts during the treatment process. Emotional reactions included disappointment, restlessness, irritability, stress, and emotional exhaustion. For several women, unmet expectations about treatment outcomes contributed to feelings of frustration and loss of motivation.

*“I got tired of this vicious cycle. I’ve been trying to regulate my periods since I was young, but it’s not working anymore, so I told myself, ‘Let it be like this.’” (P2)*

Some participants reported heightened restlessness and anxiety linked to ongoing symptoms and uncertainty about their bodies, particularly in relation to unpredictable bleeding.

*“Since my periods were irregular, I didn’t know when it would happen again. The possibility of suddenly bleeding outside scared me.” (P1)*

Irritability and mood changes were also described as treatment-related psychological effects, sometimes attributed to hormonal medication.

*“When I started taking the medication, my irritability increased. I felt more intolerant, so I stopped using it after three months.” (P3)*

Collectively, these accounts highlight that changes experienced during treatment were not limited to symptom modification but extended into women’s emotional lives, influencing their adherence, coping capacity, and overall engagement with the treatment process (Figure 2).

### 3.3 PCOS management

**Figure 3:**
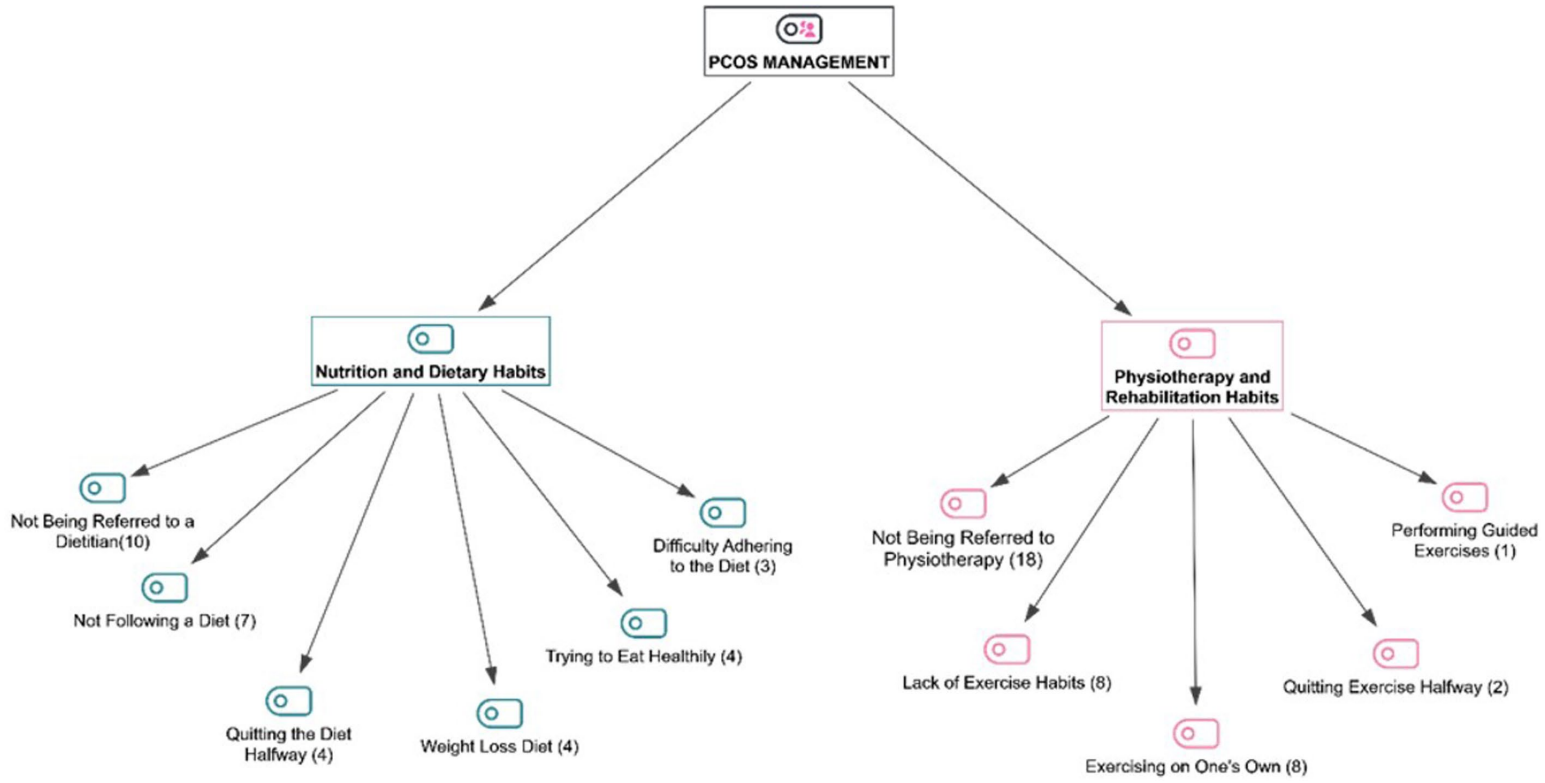
Hierarchical code-subcode model of PCOS management.

Participants described PCOS management largely as a self-directed process shaped by limited professional guidance. Most women reported managing diet and physical activity independently, often without structured support from healthcare professionals (Figure 3).

#### 3.3.1 Nutrition and dietary habits

Many participants stated that they were not referred to a dietitian and attempted to manage their condition through self-initiated dietary changes.

*“I didn’t go to a dietitian, but I was always doing diets on my own fasting diets, one day zucchini, one day water diets.” (P10)*

Some women reported making dietary modifications based on informal information sources.

*“I didn’t go to a dietitian, but I started choosing healthier foods that I heard were beneficial.” (P11)*

Even among those who consulted a dietitian, maintaining dietary changes was commonly described as challenging.

*“I tried calorie-reduction diets, intermittent fasting, and elimination diets, but I couldn’t maintain them.” (P15)*

#### 3.3.2 Physiotherapy and exercise habits

Most participants reported not being referred to physiotherapy or receiving specific exercise guidance related to PCOS (Figure 3).

*“My doctor never referred me to physiotherapy. I’ve never done exercise specifically for PCOS.” (P16)*

Exercise practices were often irregular and discontinued over time.

*“I exercise from time to time, but not regularly.” (P13)*

Some participants, however, described positive experiences when engaging in regular physical activity.

*“I started walking regularly. Exercise helped me, especially with my mood.” (P17)*

### 3.4 Challenges encountered during the PCOS process

**Figure 4:**
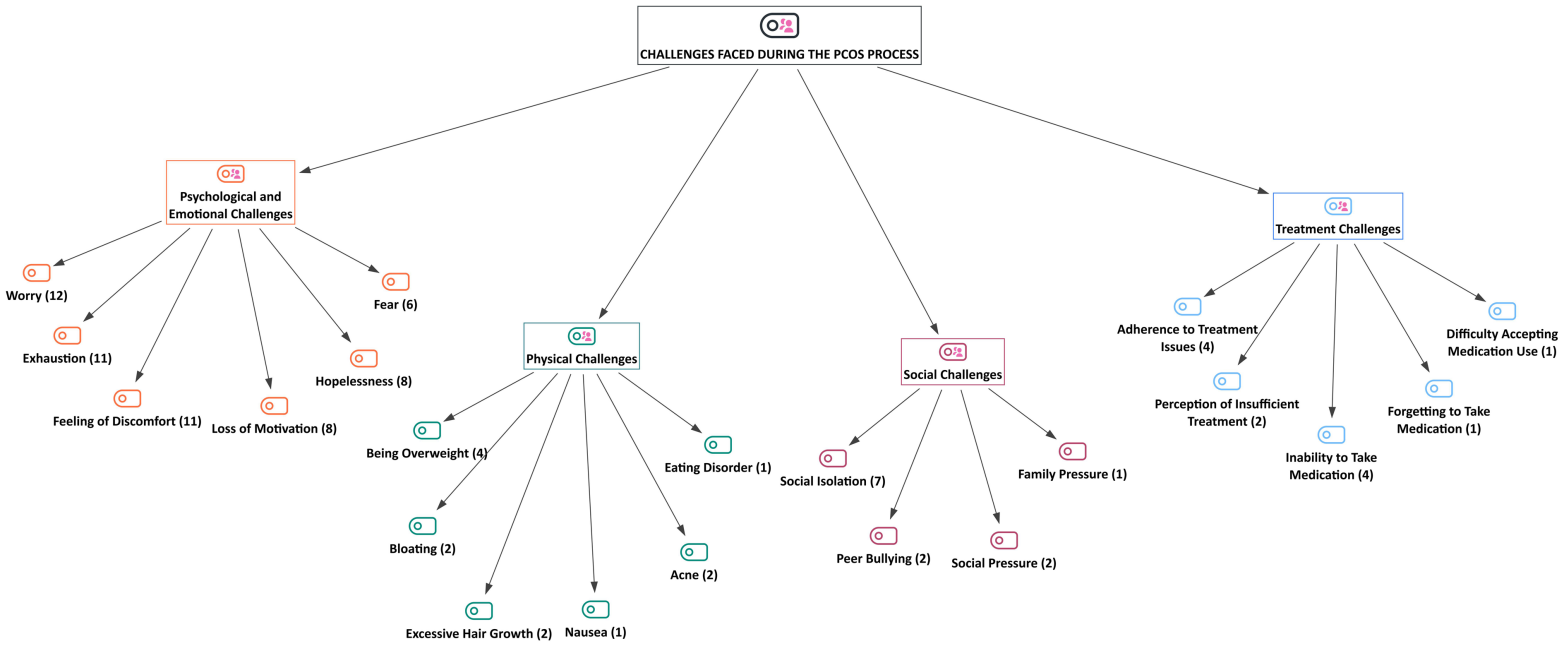
Hierarchical code-subcode model of challenges encountered in the PCOS process.

The participants’ reports on the main theme of challenges encountered during the PCOS process were Participants described multiple challenges during the PCOS process encompassing psychological, physical, social, and treatment-related difficulties. These challenges were often interrelated and shaped women’s daily lives, emotional well-being, and engagement with treatment (Figure 4).

#### 3.4.1 Psychological and emotional challenges

Participants frequently reported psychological and emotional difficulties, including anxiety, emotional exhaustion, hopelessness, loss of motivation, fear, and disturbances in body image. Many women described persistent emotional strain related to symptom burden and uncertainty about treatment outcomes (Figure 4).

*“I can’t adapt to the treatment. My stress is extremely high, and sometimes I’m afraid I won’t be able to have a child.” (P4)*

Several participants linked emotional distress to feelings of hopelessness and decreased motivation, particularly when treatment efforts did not lead to visible improvement.

*“Feeling like there’s no definitive cure drives me into hopelessness and makes it harder to follow treatment.” (P16)*

#### 3.4.2 Physical challenges

Physical challenges commonly reported by participants included being overweight, excessive hair growth, acne, bloating, nausea, and disordered eating patterns. Some women described weight-related changes as distressing, particularly when these changes limited their clothing choices or heightened body dissatisfaction.

*“I go to stores and the clothes don’t fit because I’m overweight, which makes me very unhappy.” (P4)*

Excessive hair growth and acne were frequently reported as particularly distressing physical symptoms.

*“Having hair makes me lose confidence. I can’t wear what I want.” (P10)*

#### 3.4.3 Social challenges

Social challenges included social isolation, peer bullying, family pressure, and broader social stigma. Several participants reported withdrawing from social interactions due to appearance-related concerns.

*“I stayed at home more and didn’t want to socialize.” (P2)*

Some women described experiencing ridicule related to visible symptoms such as facial hair.

*“The hair on my face became a joke among my male friends.” (P6)*

Family influence also emerged as a challenge for some participants, particularly when family members minimized the need for treatment.

*“My family said there was no need for treatment, which made the process harder for me.” (P5)*

#### 3.4.4 Treatment-related challenges

Participants described difficulties related to treatment adherence, including forgetting to take medication, resistance to medication use, and perceptions that treatment focused only on symptoms rather than providing a holistic approach (Figure 4).

*“The biggest challenge for me was having to take medication but not wanting to take it.” (P16)*

Some participants perceived treatment as insufficient due to its symptom-oriented focus.

*“They focused only on symptoms and didn’t take a holistic approach.” (P14)*

### 3.5 PCOS symptoms and coping strategies

**Figure 5:**
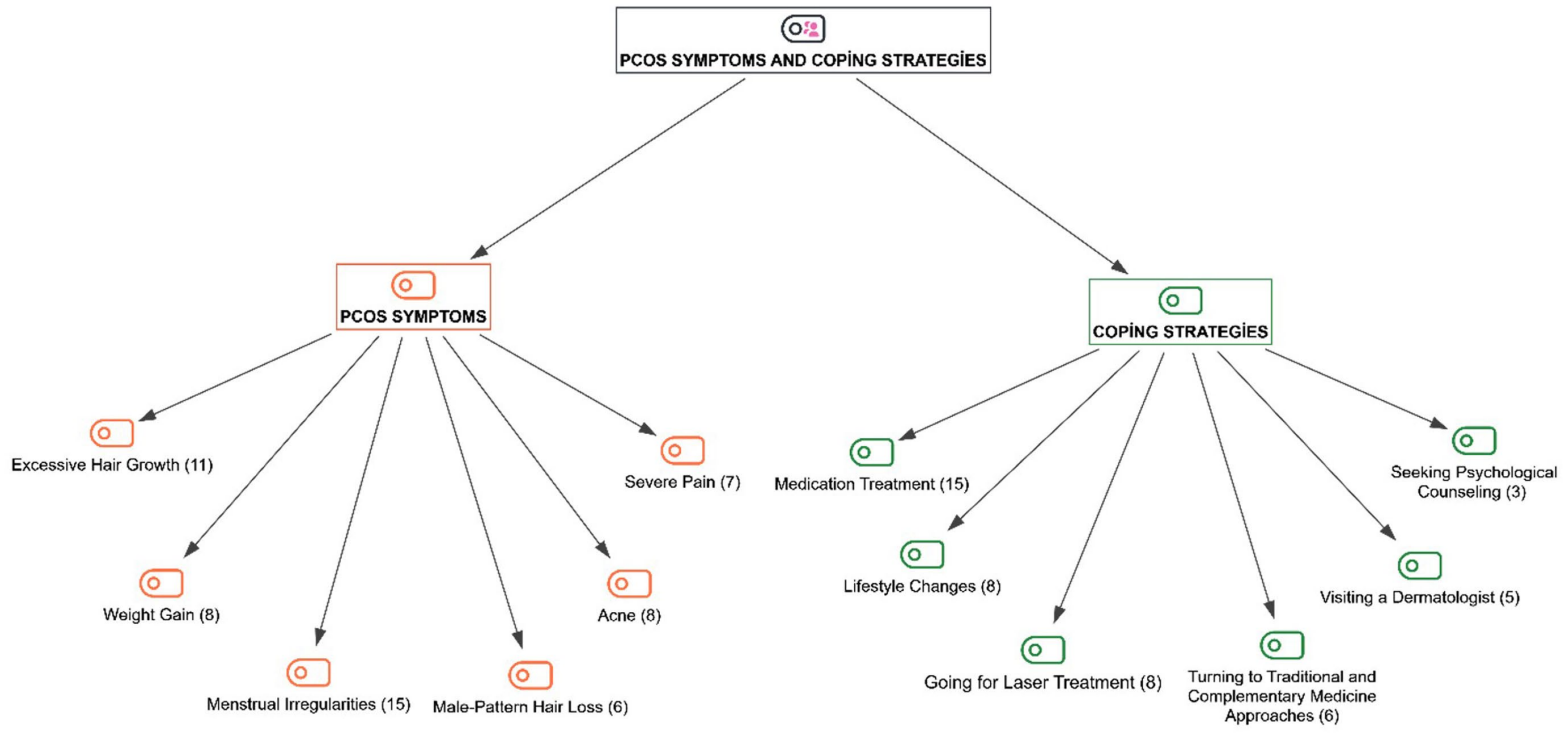
Hierarchical code-subcode model of PCOS symptoms and coping strategies.

Participants described a range of physical and psychological symptoms associated with PCOS, which substantially affected their daily lives and well-being. To manage these symptoms, women reported using various medical, lifestyle-based, cosmetic, and supportive coping strategies, often in combination (Figure 5).

#### 3.5.1 PCOS symptoms

Commonly reported symptoms included excessive hair growth, weight gain, menstrual irregularities, acne, severe menstrual pain, fatigue, and mood changes. Several participants emphasized that certain symptoms, particularly pain and visible manifestations, were especially disruptive (Figure 5).

*“I experience severe menstrual pain, to the point of fainting. On the first day, I go to the emergency room to get intravenous treatment.” (P10)*

Visible symptoms such as hirsutism and acne were frequently described as distressing and closely linked to reduced self-confidence.

*“I use medication for irregular menstruation and excessive hair growth.” (P2)*

Participants also reported persistent symptoms despite treatment, contributing to prolonged discomfort.

*“I still have skin problems, and my menstrual irregularities continue.” (P19)*

#### 3.5.2 Coping strategies

To cope with PCOS-related symptoms, participants reported employing diverse strategies. Medical treatments, particularly hormonal medications and analgesics, were commonly used (Figure 5).

*“I use oral contraceptives so that I can menstruate.” (P11)*

Lifestyle modifications, including physical activity and dietary adjustments, were also frequently reported.

*“To cope with weight gain and mood changes, I do regular reformer Pilates.” (P15)*

Cosmetic interventions, especially laser treatment, were commonly used to manage excessive hair growth, although some women expressed dissatisfaction with their long-term effectiveness.

*“I went for laser treatment, but the hair still grows back.” (P16)*

Some participants turned to traditional or complementary approaches such as herbal remedies, cupping, or leech therapy.

*“As an alternative, I tried cupping and leech therapy, but they didn’t work.” (P18)*

In addition, a small number of participants sought psychological or psychiatric support to address emotional difficulties associated with PCOS.

*“I regularly receive psychological support for mood-related problems.” (P13)*

Overall, coping strategies were often characterized by trial-and-error approaches and reflected women’s efforts to manage both physical symptoms and emotional challenges associated with PCOS (Figure 5).

### 3.6 Uncertainties and concerns

Participants expressed ongoing uncertainties and concerns related to PCOS, particularly regarding long-term health outcomes, reproductive potential, symptom management, and psychological well-being. These uncertainties were described as persistent and were often accompanied by feelings of anxiety and insecurity about the future.

#### 3.6.1 Lack of knowledge

Many participants reported difficulties in accessing clear, reliable, and consistent information about PCOS and its management. Unanswered questions frequently concerned hormone levels, stress management, sexual health, and how to effectively control symptoms.

*“I still don’t know how I should manage PCOS properly.” (P2)*

Some women described confusion arising from inconsistent or contradictory information obtained from healthcare providers, social media, or informal sources.

*“I don’t know whether the information I read on social media is accurate.” (P15)*

#### 3.6.2 Concerns about the future

Concerns about the future were predominantly related to fertility, disease progression, and the long-term consequences of PCOS. Many participants expressed anxiety about their ability to conceive.

*“I’m worried about whether I’ll be able to have a child.” (P1)*

Uncertainty was also reported regarding the persistence of symptoms, the effectiveness of treatment, and the possibility of complete recovery.

*“I keep wondering if I will ever fully get rid of this condition.” (P16)*

Some participants described broader health-related fears, including concerns about developing other gynecological conditions.

*“I’m afraid PCOS could lead to more serious diseases in the future.” (P10)*

Participants’ uncertainties and concerns were further reflected in the questions they reported being unable to find answers to. These questions, summarized in Table 2, primarily focused on fertility, long-term disease outcomes, symptom management, psychological well-being, and the effectiveness of treatment.

**Table 2:** Participants’ questions and concerns.

Does active sexuality positively affect PCOS?	Why does not my bloating go away?
How and to what extent are my hormones affected?	Will I completely get rid of this disease?
How effective is diet in PCOS?	How should I manage PCOS?
What causes my vaginal discharge?	Will I have a child?
How does my abdominal pain go away?	Will my psychological problems end?
How does my stress go away?	Will I be able to get rid of the hair?
What awaits me in my life?	Will my treatment have positive results?
How long will the treatment continue?	Will my acne go away?
Do I need to exercise?	Will I get better if I give birth?
Could PCOS be the reason I cannot lose weight?	Will I get cancer?
Would it be effective if I used the medication regularly?	Will I be dependent on medication forever?
How will I manage these symptoms?	Will my routine be disrupted if I stop dieting and exercising?
How can I cope with the emotional difficulties I experience?	Will PCOS trigger different women’s diseases?
Is the information written on social media true?	Is PCOS genetic, will my child have it in the future?
Why cannot I lose weight?	

## 4 Discussion

This study examined the challenges women with PCOS experience across the processes of diagnosis and management. Although many participants initially accepted the diagnosis, they frequently reported uncertainty regarding treatment decisions and difficulties accessing consistent care. Commonly reported symptoms included menstrual irregularities, hirsutism, and weight related concerns, while coping strategies involved medication use, lifestyle changes, and cosmetic practices. Together, these findings highlight the multi layered nature of living with PCOS and the ways clinical, emotional, and informational factors intersect over time.

### 4.1 Diagnosis of PCOS

PCOS is a complex condition involving heterogeneous physiological, metabolic, and endocrine features. Variability in diagnostic criteria and their application in clinical practice has been widely discussed in the literature and has been associated with delays and inconsistencies in diagnosis. Although the Rotterdam criteria introduced standardized phenotypic classification in 2003, more recent international guidelines emphasize an evidence-based and individualized approach that accounts for age, ethnicity, metabolic risk, and long-term outcomes, moving away from rigid phenotype categorizations (6). Despite these developments, previous research suggests that women often require consultations with multiple healthcare professionals and may experience prolonged diagnostic timelines, with an average diagnostic delay of approximately one year (15).

Consistent with these reports, women in the present study frequently described confusion and uncertainty during the diagnostic process. Participants attributed these experiences to inconsistent medical advice and limited access to standardized information, findings that align with those of Gibson-Helm et al., who reported low satisfaction with diagnostic experiences among women with PCOS (7). Similarly, Copp et al. found that diagnosis may evoke mixed emotional responses, including fear, embarrassment, and relief (13). Other studies have also shown that receiving a PCOS diagnosis can trigger anxiety, hopelessness, and, in some cases, disengagement from treatment (12, 16).

Importantly, the present findings suggest that challenges during diagnosis extend beyond clinical uncertainty to encompass communication and relational aspects of care. Perceived inadequacies in information provision and limited opportunities for dialogue appeared to contribute to emotional distress and uncertainty regarding long-term management. Previous studies have indicated that prolonged or unclear diagnostic processes may have broader implications for reproductive planning and psychosocial well-being (17).

Psychological support has been identified in the literature as an important component of PCOS care, particularly in addressing emotional responses associated with diagnosis and ongoing symptom management (18, 19). In this context, the findings of the present study underscore the relevance of considering psychological dimensions alongside biomedical criteria during the diagnostic phase.

Overall, these findings highlight that PCOS diagnosis is experienced not merely as a clinical event but as a complex and emotionally laden process. Approaches that emphasize clear communication, timely diagnosis, and multidisciplinary collaboration may play an important role in shaping women’s early experiences of PCOS and their subsequent engagement with care (6).

### 4.2 Changes experienced during the treatment process

The heterogeneous and variable nature of PCOS has been widely recognized, and treatment approaches often require the combination of pharmacological and nonpharmacological strategies tailored to individual needs (20). In addition to managing oligoovulation and hyperandrogenism, previous studies emphasize the importance of addressing metabolic health, psychological well being, and quality of life as integral components of PCOS management. For instance, Jungari et al. (21) reported that combined interventions involving medication and lifestyle modification led to symptom improvement in women with PCOS, a sample largely characterized by overweight status at baseline and high rates of menstrual irregularity.

Despite these reported benefits, findings regarding treatment related psychological outcomes remain inconsistent across the literature. Some small scale studies have suggested that PCOS treatment may be associated with improvements in depressive symptoms, particularly when weight management and hormonal regulation are achieved (22–24). However, other investigations have found no significant changes in anxiety or depression levels following treatment, highlighting the variability of psychological responses and the complex interplay between symptoms, expectations, and treatment experiences (25).

In line with these mixed findings, participants in the present study described treatment related experiences marked by emotional distress, including confusion, disappointment, and symptoms of stress or low mood. Difficulties in understanding treatment options and managing expectations appeared to contribute to these responses. Hoyos et al. similarly reported that more than half of women with PCOS were dissatisfied with the information they received regarding treatment alternatives, underscoring the potential impact of inadequate communication on treatment experiences (26).

Taken together, these findings suggest that changes experienced during PCOS treatment extend beyond physiological outcomes and involve significant emotional and informational dimensions. Rather than reflecting a linear process of symptom improvement, treatment was often perceived as a fluctuating and uncertain journey, shaped by inconsistent responses and unmet expectations. Understanding treatment related changes from this multidimensional perspective may be important for interpreting women’s engagement with care and their longer term management experiences.

### 4.3 PCOS management

Both pharmacologic and nonpharmacologic treatments are essential for effective PCOS management. Among nonpharmacologic strategies, lifestyle modifications including regular exercise and individualized nutritional interventions play a critical role (27). Although no universal treatment exists, dietary improvements and increased physical activity have been associated with better PCOS outcomes (28, 29). Findings regarding diet and exercise patterns in women with PCOS are mixed. Some studies reported no significant differences in nutrient intake, diet quality, or physical activity levels between women with and without PCOS (30). Moran et al. reported better diet quality among women with PCOS (31), although physical activity levels remained similar (32).

In our study, most participants had not been referred to a dietician or physiotherapist; seven reported not following any diet, and eight had inadequate exercise habits. While some evidence suggests that women may adopt healthier behaviors after diagnosis, the literature remains inconclusive. Longitudinal research is needed to determine whether these changes are sustained over time.

### 4.4 Challenges faced during the diagnosis process, PCOS symptoms, and coping strategies

Given the lifelong nature of PCOS, an effective patient–provider relationship plays a central role in disease management. Clear and consistent communication has been associated with informed decision making and improved treatment outcomes (7, 33). However, consistent with earlier studies, the present findings highlight persistent challenges in patient–provider interactions, including delayed diagnosis, insufficient information sharing, and communication difficulties, which contributed to frustration, emotional distress, and reduced treatment adherence (12, 34). Participants particularly emphasized difficulties in accessing clear information regarding long term management, a finding that aligns with previous reports demonstrating how inadequate communication may hinder effective self management and sustained engagement with care (35, 36).

Women in this study commonly reported symptoms such as hirsutism, acne, menstrual irregularities, and weight related concerns, all of which are known to impair self esteem, social participation, and psychological well being (12, 14). As PCOS affects multiple systems, including metabolic, reproductive, and dermatologic health, its impact on quality of life has been described as comparable to that observed in chronic conditions such as diabetes (37). While pharmacological approaches such as metformin are frequently used to support lifestyle interventions, uncertainty remains regarding their broader long term effects, including potential intergenerational outcomes (38). Together, these findings underscore the complexity of PCOS management and the importance of individualized and multidisciplinary approaches to address both clinical and psychosocial needs (6).

Coping with PCOS involves cognitive and behavioral efforts to manage stress and symptom burden. According to the transactional model of stress and coping, strategies may be broadly categorized as active or passive, with coping styles influencing both psychological and physiological responses (39). In this study, most women primarily adopted active coping strategies, including medication use, lifestyle modifications, and cosmetic procedures such as laser therapy. A smaller number also reported the use of complementary approaches. These patterns are consistent with earlier findings indicating that women with PCOS employ diverse coping strategies in an effort to manage symptoms and enhance overall well being (40).

A key contributor to ongoing uncertainty identified in this study was the lack of accessible and comprehensive information regarding PCOS and its long term management. Limited patient education and insufficient support resources have previously been shown to exacerbate uncertainty and emotional burden (6). Although digital health tools such as the ASKPCOS application provide evidence based information and self management support internationally, the absence of culturally and linguistically adapted resources, including a Turkish version, highlights a continued unmet need in this context (41).

### 4.5 Strengths and limitations of the study

One of the strengths of this study lies in its qualitative design, which enabled an in depth exploration of women’s experiences of PCOS across diagnostic, treatment, and management processes. The use of semi structured interviews allowed participants to articulate their experiences in their own words, providing rich and nuanced insight into the emotional, social, and clinical dimensions of living with PCOS. In addition, the multidisciplinary composition of the research team supported a holistic interpretation of the findings, incorporating perspectives related to physical health, lifestyle management, and psychosocial wellbeing.

This study also has several limitations that should be acknowledged. This study provides clinically oriented insights into women’s experiences with PCOS; however, its primary focus was on diagnosis, treatment, and management-related challenges. Therefore, the broader sociocultural and socio-psychological dimensions of these experiences were not explored in depth. In particular, gendered stigma, family expectations, and the wider cultural meanings attached to fertility, body image, and femininity in the Turkish context represent important areas for further investigation. Future research should adopt more interpretive and theoretically informed qualitative approaches to better understand how sociocultural norms and social structures shape women’s lived experiences of PCOS. Such efforts may contribute to a more comprehensive understanding of the condition and support the development of culturally sensitive healthcare practices. As a qualitative study with a relatively small sample size, the findings may not be generalizable to all women with PCOS. Instead, the results aim to provide analytical depth rather than representativeness. Furthermore, although efforts were made to encourage open-ended discussion, one or two interview questions contained leading elements, which may have influenced the direction of some responses. To mitigate this limitation, participants were consistently encouraged to elaborate freely and to introduce issues they considered personally meaningful during the interviews.

Despite these limitations, the study offers valuable insights into how women experience PCOS within a particular sociocultural context and highlights areas that may inform future qualitative research and practice oriented investigations.

## 5 Conclusion

This study underscores the substantial challenges women encounter across the PCOS diagnostic and treatment journey, including delayed diagnosis, limited access to accurate information, and variability in management approaches. Such experiences appear to contribute to heightened emotional distress, decreased engagement with treatment, and difficulties in maintaining recommended lifestyle modifications.

The findings highlight the critical need for patient-centered care characterized by clear communication, individualized management plans, and integrated psychological support. Enhancing healthcare providers’ awareness of PCOS and improving access to multidisciplinary services—including medical, nutritional, and psychological interventions—may strengthen women’s capacity to effectively manage their condition.

Although the present study did not directly measure the effectiveness of support programs, the insights gained offer valuable direction for developing structured educational, behavioral, and lifestyle-focused interventions. Future research should prioritize evaluating long-term treatment adherence, the impact of psychological support models, and the implementation of evidence-based lifestyle strategies aligned with contemporary international PCOS guidelines.

## Data Availability

The original contributions presented in the study are included in the article/supplementary material, further inquiries can be directed to the corresponding author.
